# QiShenYiQi pill improves the reparative myocardial fibrosis by regulating autophagy

**DOI:** 10.1111/jcmm.15695

**Published:** 2020-09-02

**Authors:** Shichao Lv, Peng Yuan, Jianping Dong, Chunmiao Lu, Meng Li, Fan Qu, Yaping Zhu, Zhuo Yuan, Junping Zhang

**Affiliations:** ^1^ First Teaching Hospital of Tianjin University of Traditional Chinese Medicine Tianjin China; ^2^ Tianjin Key Laboratory of Traditional Research of TCM Prescription and Syndrome Tianjin China; ^3^ Health Center of Balitai Town Tianjin China; ^4^ Jiashan Hospital of Traditional Chinese Medicine Jiaxing China

**Keywords:** autophagy, PI3K/Akt‐mTOR pathway, reparative myocardial fibrosis, traditional Chinese medicine

## Abstract

QiShenYiQi pill (QSYQ), a traditional Chinese medicine, is well known for improving the myocardial remodelling, but the dose‐effect relationship of its intervention in the reparative myocardial fibrosis is still unclear. We investigated the effect of QSYQ on the reparative myocardial fibrosis in cardiac myosin‐induced rats and explored its mechanism of action by regulating autophagy. The results indicated that QSYQ increased LVEF and LVFS, and decreased the LVEDD, LVESD, HMI, LVMI, myocardial inflammation histology score, and collagen volume fraction in a dose‐dependent manner. In addition, QSYQ declined the number of autophagosomes, down‐regulated the expression of myocardial Beclin‐1 and LC3B, up‐regulated the expression of myocardial p62 and increased the ratios of myocardial p‐PI3K/PI3K, p‐Akt/Akt and p‐mTOR/mTOR. We provided evidence for that QSYQ could inhibit excessive myocardial autophagy by regulating the PI3K/Akt‐mTOR pathway and can be a potential therapeutic approach in treating the cardiovascular diseases such as myocarditis and dilated cardiomyopathy.

## 1 INTRODUCTION

Myocardial fibrosis is characterized by the metabolic imbalance of collagen synthesis and metabolism, which leads to pathological change of myocardial remodelling, and mostly seen in various cardiovascular diseases.^1^ Myocardial fibrosis is categorized into reactive fibrosis and reparative fibrosis.^2^ Reactive fibrosis occurs in the myocardial hypertrophy caused by excessive heart pressure without myocardial cell loss, whereas reparative fibrosis occurs in acute myocardial ischaemia and dilated cardiomyopathy. Both can affect the myocardial collagen content and promote myocardial diastolic and/or contractile dysfunction. Reparative myocardial fibrosis is the pathological basis for the development of myocarditis and post‐inflammatory dilated cardiomyopathy to refractory heart failure, promoting the deterioration of the diseased heart function in dilated myocardium.^3^ Therefore, reversing myocardial remodelling might add value to the treatment of dilated cardiomyopathy.^4^ Dilated cardiomyopathy is complex heterogeneous cardiomyopathy characterized by ventricular enlargement and reduced myocardial contractile function, with a 5‐year mortality rate of 15%‐50%.^5^ Among all the factors such as infection, autoimmunity, cellular immunity and genetics, autoimmune reactions mostly induce the myocarditis and dilated cardiomyopathy.^6^ Accumulating evidence has demonstrated that Lewis rats induced by cardiac myosin can develop autoimmune myocarditis, which is gradually progressed into dilated cardiomyopathy.^7^, ^8^

QiShenYiQi pill (QSYQ) is a traditional Chinese medicine composed of *Radix Astragali*, *Radix Salviae Miltiorrhizae*, *Radix Notoginseng* and *Lignum Dalbergiae Odoriferae*. QSYQ is approved by the China State Food and Drug Administration in 2003 for the treatment of cardiovascular disease. Many studies have reported that QSYQ can alleviate myocardial ischaemia/reperfusion (I/R) injury,^9^, ^10^ inhibit myocardial fibrosis caused by pressure overload^11^, ^12^, ^13^ and delay ventricular remodelling caused by ligation of anterior descending coronary artery.^14^, ^15^, ^16^ Moreover, QSYQ can improve myocardial injury induced by doxorubicin,^17^, ^18^ regulate myocardial collagen metabolism in experimental autoimmune myocarditis^19^, ^20^ and prevent high glucose‐induced H9c2 myocardial cell damage.^21^ In the current study, we investigated the effect of QSYQ on the reparative myocardial fibrosis in the cardiac myosin‐induced rats and explored its mechanism of action by regulating autophagy.

## 2 METHODS AND MATERIALS

### 2.1 Animals

Male Lewis rats, SPF grade, weighing 220‐250 g, were purchased from Beijing Vital River Laboratory Animal Technology Co., Ltd. (Beijing, China; certificate No. SCXK(Jing)2016‐0011). Animals were kept under standard conditions with a mean temperature of 22°C ± 2°C, a mean relative humidity of 55% ± 10 and a defined day‐and‐night‐rhythm of 12 hours light and 12 hours dark, and ate water freely. All animal procedures performed in this study were approved by the Animal Ethics Committee of Tianjin University of Traditional Chinese Medicine of China (No. TCM‐LAEC2016016) and were in compliance with the Guide for the Care and Use of Laboratory Animals released by the US National Institutes of Health (NIH Publication No. 85‐23, revised 1996).

### 2.2 Materials

All reagents in the study are as follows: QiShenYiQi pill (Tasly Pharmaceutical Co., Ltd., Tianjin, China); 3‐methyladenine (ApexBio, Houston, TX, USA); porcine cardiac myosin and complete freund's adjuvant (Sigma Aldrich, USA); haematoxylin and eosin staining kit, masson trichrome staining kit, RIPA lysate, phosphatase inhibitor and protease inhibitor (Leagene Biotechnology Co., Ltd., Beijing, China); BCA protein concentration test kit (Boster Biological Technology Co., Ltd., Wuhan, China); PI3K antibody, Akt antibody, mTOR antibody, LC3B antibody, Beclin‐1 antibody, p62 antibody, HRP‐conjugated Affinipure Goat Anti‐Mouse IgG, HRP‐conjugated Affinipure Goat Anti‐Rabbit IgG and ECL chemiluminescence detection kit (Proteintech Group, Inc, USA); p‐PI3K antibody (Abcam, Cambridge, UK); p‐akt antibody and p‐mTOR antibody (Cell Signaling Technology, Inc, USA); RNA extraction kit (Takara Biomedical Technology Co., Ltd., Beijing, China); TransScript First‐Strand cDNA Synthesis SuperMix (TransGen Biotech Co., Ltd., Beijing, China); PowerUp™ SYBR™ Green Master Mix (Thermo Fisher Scientific, Inc, USA).

### 2.3 Establishment of a reparative myocardial fibrosis rat model

We used the injection of cardiac myosin to induce experimental autoimmune myocarditis and established the rat models of reparative myocardial fibrosis.^22^ Porcine cardiac myosin with a concentration of 6.4 mg/mL was mixed with an equal volume of Freund's complete adjuvant to prepare a thick water‐in‐oil emulsion. Lewis rats were placed on an adaptive feeding routine for 3‐5 days and then injected with the mixed emulsion subcutaneously on the lower extremity foot pads on day 0 and day 7. Later, 0.3 mL of emulsion, equivalent to 1 mg of porcine cardiac myosin, was injected into each of the rats.^23^ The control group was injected with a mixture of an equal volume of phosphate buffer and complete Freund's adjuvant.

### 2.4 Animal grouping and administration

At 4 weeks after the initial injection of cardiac myosin, the rats were randomly divided into 5 groups: (1) model group (equal volume of distilled water by gavage, n = 8), (2) the 3‐methyladenine group (15 mg/kg, intraperitoneal injection, n = 8), (3) QSYQ low‐dose group (135 mg/kg by gavage, n = 8), (4) QSYQ medium‐dose group (270 mg/kg by gavage, n = 8), (5) QSYQ high‐dose group (540 mg/kg by gavage, n = 8), (6) the control group using normal rats (equivalent volume of distilled water by gavage, n = 8). 3‐methyladenine (3‐MA) is widely used as an autophagy inhibitor through inhibiting class III phosphoinositide 3‐kinase (PI3K).^24^ QSYQ in combination with distilled water was administered as a solution through oral gavage, and then, doses were calculated according to the body surface area.^25^, ^26^

### 2.5 Assessment of cardiac structure and function via echocardiography

Echocardiography was performed 4 weeks later after drug intervention. After intraperitoneal anaesthesia using 3% pentobarbital sodium (45 mg/kg), each rat was placed in a supine position on the operating table, and the skin in front of the chest was prepared, and then the coupling agent was applied on it. Vevo^®^ 2100 ultra‐high resolution small animal ultrasound imaging system (probe MS‐250, frequency 21 MHz) was used to obtain a long‐axis section image of parasternal left ventricle under a two‐dimensional mode (B‐Mode). The sampling line was placed at a position of the maximum diameter of the left ventricle to display the M‐type ultrasound image and measured using a long‐axis measurement package (PLAX). Each index was measured for 3 cardiac cycles to calculate the mean value.

### 2.6 Morphological observation of the heart

**FIGURE 1:**
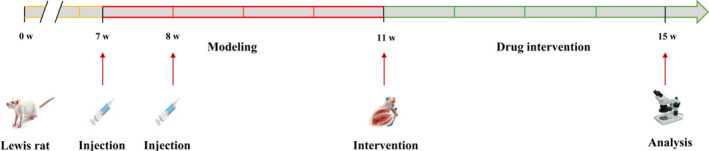
The flow diagram of experiment

The body weight (BM) of rats was weighed again before sampling. After anaesthesia, blood was quickly taken from the femoral artery by centrifugation (1609.92 *g*, 10 minutes), serum collection and aliquot. Then, the chest was opened, the heart was taken out, and the blood was washed off with normal saline at 4°C. The filter paper was used for drying, and the heart morphology and surface lesions were observed. The tissues and great vessels around the heart were removed, and the heart mass (HM) was weighed to calculate the heart mass index (HMI, HMI = HM/BM (mg/g)). Then, the left and right atria along with the free wall of right ventricle were removed, and the left ventricle and interventricular septum were preserved. The left ventricular mass (LVM) was weighed to calculate the left ventricular mass index (LVMI), LVMI = LWH/BM (mg/g) (Figure 1).

### 2.7 Pathological staining to observe the cardiac tissue morphology

The myocardial tissues were fixed by 4% paraformaldehyde, and conventional ethanol was used for gradient dehydration, transparency, paraffin embedding and continuous sections (5 μm thick). H and E staining and Masson staining were performed followed by sealing with neutral gum, and the changes of myocardial histomorphology were observed under a light microscope. Then, five microscopic fields were selected randomly from each section to calculate the inflammatory cell infiltration and the collagen volume fraction (CVF, CVF = myocardial collagen fibre area/total image area). According to HE staining, the histological grade of myocardial inflammation tissue was carried out^27^: Grade 1, scattered focal inflammatory lesions; Grade 2, multiple isolated inflammatory lesions; Grade 3, diffused inflammation invading the epicardium; Grade 4, diffused inflammation invading the epicardium with focal transmural inflammation; Grade 5, diffused inflammation with necrosis.

### 2.8 Observation of myocardial autophagosomes by transmission electron microscope

After fixation with 3% glutaraldehyde solution and 1% osmic acid, myocardial tissues smaller than 1 mm^3^ were dehydrated with gradient ethanol and embedded with Epon812. Then, the semi‐thin sections underwent aniline blue staining. After semi‐thin sections positioning under the light microscope, ultra‐thin sections were prepared by double staining with uranyl acetate and lead citrate. Then, the myocardial autophagosomes and its ultrastructure were observed under a transmission electron microscope.

### 2.9 Real‐time quantitative PCR detection of myocardial target gene expression

Total RNAs were extracted from the myocardial tissue samples with RNAiso Plus reagent, and the concentration of RNA was measured by an ultra‐micro nucleic acid protein analyser. Then, it was reverse transcribed to cDNA, and the cDNA was used as a template to carry out the RT‐qPCR reaction with SYBR Green under the following reaction conditions: pre‐denaturation at 94℃ for 30 seconds; denaturation at 94℃ for 5 seconds, annealing, and extension at 60℃ for 30 seconds, for 40 cycles. Following the results of RT‐qPCR detection, the 2^−ΔΔCt^ method was performed for relatively quantitative data analysis. The primers were designed and synthesized by Shanghai Biotechnology Co., Ltd. The forward Beclin‐1 primer was 5′‐TGTTTGGAGATGTTGGAGCA‐3′, and the reverse Beclin‐1 primer was 5′‐ATGGAAGGTCGCATTGAAGA‐3′. The forward LC3B primer was 5′‐CGGAGCTTCGAACAAAGAGTG‐3′, and the reverse LC3B primer was 5′‐CTTGGTCTTGTCCAGGACGG‐3′. The forward p62 primer was 5′‐GGAGACCCCAAATATGCCC‐3′, and the reverse p62 primer was 5′‐CAGACACCCCACGACCACGAGAGGG‐3′. The forward GAPDH primer was 5′‐AGATGGTGAAGGTCGGTGTG‐3′, and the reverse GAPDH primer was 5′‐CTGGAAGATGGTGATGGGTT‐3′.

### 2.10 Western blot detection of target protein expression in rat myocardium

The moderate myocardial tissue samples were added into the lysis liquid of protein and pulverized by the ultrasonic disrupter. After lysing for 30 minutes on ice and centrifuging at 4℃ and 9659.52 *g* for 10 minutes, the protein concentration was measured by the BCA method. The protein was separated using sodium dodecyl sulphate‐polyacrylamide gel electrophoresis (SDS‐PAGE) and transferred to polyvinylidene fluoride (PVDF) membranes. The PVDF membrane was incubated for 2 hours in 5% skim milk powder. Primary antibodies including p‐PI3K antibody (dilution 1:1000), PI3K antibody (Dilution 1:1000), p‐Akt (dilution 1:2000), Akt (dilution 1:500), p‐mTOR (dilution 1:1000), mTOR (dilution 1:2000), LC3B antibody (dilution 1:300), Beclin‐1 antibody (diluted 1:1000), and p62 antibody (diluted 1:1000), β‐actin antibody (dilution 1:5000) were added and incubated at 4°C overnight. Then, the membranes were incubated with the appropriate horseradish peroxidase (HRP)‐conjugated secondary antibody (dilution 1:5000) at room temperature for 2 hours. After the ECL luminescent reagent is coloured, the automatic gel imaging system was performed by exposure imaging. Image Lab software was used to detect the expression of protein bands, and the relative expression of the target protein was calculated using β‐actin as the internal reference (relative expression of the target protein = grey value of the target protein/grey value of the internal reference β‐actin).

### 2.11 Statistical method

SPSS 11.5 statistical software was used for all statistical analyses and the data were expressed as mean ± standard deviation ($\bar{x}\pm s$). Statistical analysis was performed using one‐way ANOVA followed by the least significant difference (LSD) test for multiple comparisons. *P* < 0.05 was set as statistically significant.

## 3 RESULTS

### 3.1 Effect of QSYQ on the structure and function of rat heart

**FIGURE 2:**
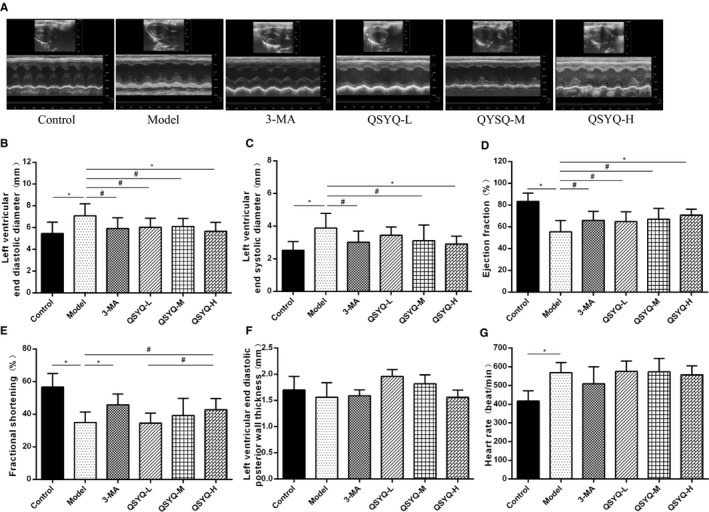
Effect of QiShenYiQi pill (QSYQ) on heart structure and function in rat. A, Echocardiography of rat heart. B, Left ventricular end diastolic diameter in rats. C, Left ventricular end systolic diameter in rats. D, Left ventricular ejection fraction in rats. E, Left ventricular fractional shortening in rats. F, Left ventricular End‐diastolic posterior wall thickness in rats. G, Heart rate in rats. Groups: control (n = 8), model (n = 8), 3‐MA (n = 8), QSYQ‐L (n = 8), QSYQ‐M (n = 8), QSYQ‐H (n = 8). Data are expressed as mean ± SD. *P < 0.01, ^#^ *P *< 0.05

To investigate the cardiac function of rats with repaired myocardial fibrosis, we used a rat model of cardiac myosin injection to induce repaired myocardial fibrosis. The results showed that the heart rate (HR), left ventricular end‐diastolic diameter (LVEDD) and left ventricular end‐systolic diameter (LVESD) increased, and the left ventricular ejection fraction (LVEF) and left ventricular fractional shortening (LVFS) decreased significantly, suggesting that it caused rats severe cardiac dysfunction. 3‐MA and QSYQ significantly increased LVEF and LVFS, indicating that treatment with 3‐MA and QSYQ can significantly improve the LV contractile function of repaired myocardial fibrosis. In addition, in rats treated with 3‐MA and QSYQ, the increase in LVEDD and LVESD was significantly weakened compared to rats injected with myocardial myosin, indicating that 3‐MA and QSYQ significantly attenuated the repaired myocardial fibrosis LV remodelling, and QSYQ showed a dose‐dependent trend, and the effect of high‐dose QSYQ was more significant (Figure 2).

### 3.2 Effect of QSYQ on the general shape of rat heart

**FIGURE 3:**
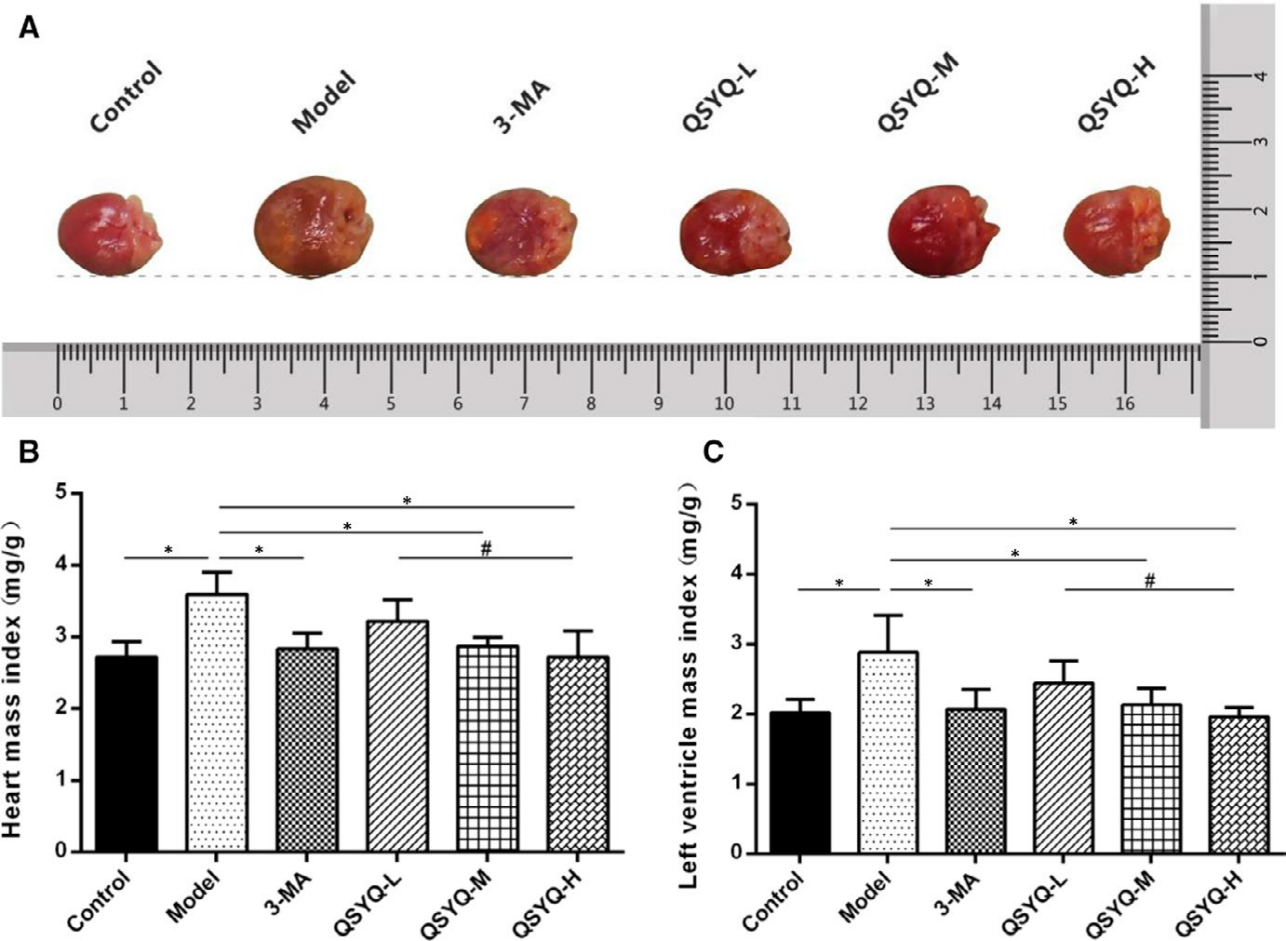
Effect of QiShenYiQi pill (QSYQ) on the general shape of rat heart. A, General morphology of rat heart. B, Heart mass index of rats. C, Left ventricular mass index of rats. Groups: control (n = 8), model (n = 8), 3‐MA (n = 8), QSYQ‐L (n = 8), QSYQ‐M (n = 8), QSYQ‐H (n = 8). Data are expressed as mean ± SD. *P<0.01, ^#^ *P *< 0.05

Compared with the control group, an enlarged heart of white infiltration spots on the surface was observed in the model group. Both HMI and LVMI were significantly increased (*P* < 0.01). In contrast with the model group, the heart disease of rats in 3‐methyladenine and QSYQ groups reduced, and both HMI and LVMI showed a decreasing trend (*P* < 0.01). The QSYQ high‐dose group had a tendency to further reduce HMI and LVMI in rats (Figure 3).

### 3.3 Effect of QSYQ on myocardial histology in rats

**FIGURE 4:**
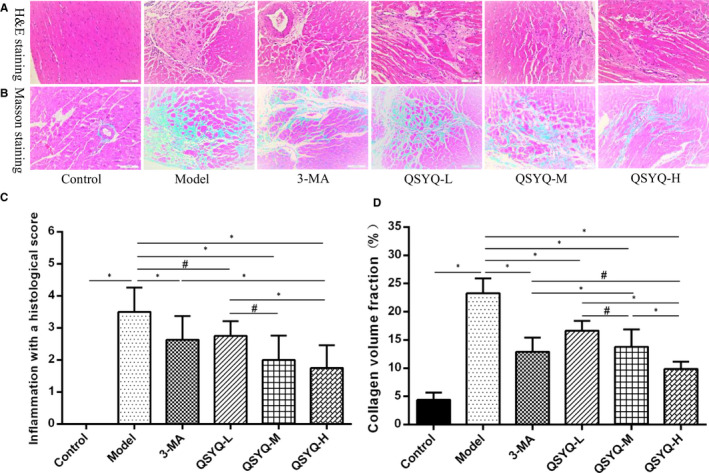
Effect of QiShenYiQi pill (QSYQ) on myocardial histology in rats. A, Representative photomicrograph of haematoxylin and eosin (H&E) staining of myocardium. B, Representative photomicrograph of mason trichrome staining of myocardium. C, The inflammation with a histological score for each group. D, The collagen volume fraction for each group. Data are expressed as mean ± SD. *P < 0.01, ^#^ *P *< 0.05

The H and E staining revealed normal morphological structure without inflammatory infiltration lesions of the myocardial tissue of the control group. Compared with the control group, the model group showed increased and infiltrated myocardial inflammatory cells with hypertrophy, accompanied by necrosis. The histological score of myocardial inflammation was significantly higher than that of the control group (*P* < 0.01), whereas the inflammatory cells of the 3‐methyladenine and QSYQ group were decreased, and the histological score of myocardial inflammation was lower than that of the model group (*P* < 0.01 or *P* < 0.05). QSYQ reduced the histological score of the myocardial inflammation dose‐dependently. Thus, the QSYQ high‐dose group further reduced myocardial inflammation histological score (*P* < 0.01 or *P* < 0.05), and the effect was better than the 3‐methyladenine group (*P* < 0.01) (Figure 4).

The Masson staining revealed a small amount of blue‐stained fibres in the myocardial tissue of the control group, without any changes to the myocardial interstitial fibrosis. Compared with the control group, a large number of blue‐stained fibres and more collagen deposition were observed in the myocardial tissue and myocardial interstitial with the disordered arrangement in the model group, respectively. The myocardial collagen volume fraction was also found to be significantly higher than the control group (*P* < 0.01). Compared with the model group, the degree of myocardial fibrosis and myocardial collagen volume fraction in the 3‐methyladenine group and the QSYQ group were reduced than the model group (*P* < 0.01). The effect of the QSYQ group on reducing the myocardial collagen volume fraction was dose‐dependent since the QSYQ high‐dose group further decreased the myocardial collagen volume fraction (*P* < 0.01 or *P* < 0.05) and exhibited better effect than the 3‐methyladenine group (*P* < 0.05) (Figure 4).

### 3.4 Effect of QSYQ on myocardial autophagy in rats

**FIGURE 5:**
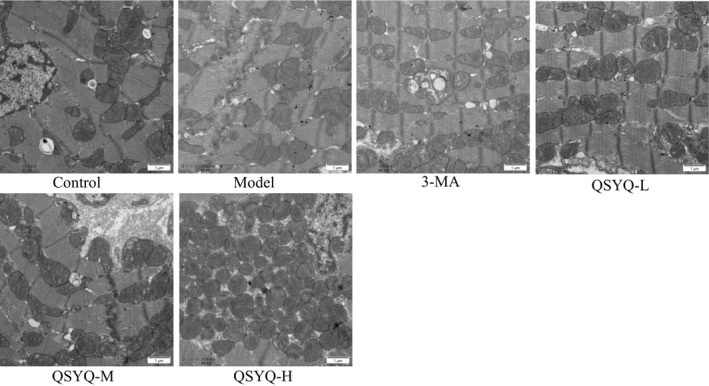
Effect of QiShenYiQi pill (QSYQ) on myocardial autophagy in rats. Transmission electron microscopy images of autophagosomes in myocardial. Arrows indicate autophagosomes

In the control group, the myocardial fibres were arranged neatly and tightly with clear stripes. The number of mitochondria was rich with clear cristae and a small number of scattered autophagosomes were also observed. In the model group, rats showed a disordered arrangement of the myocardial fibres and poor clarity stripes. Several mitochondria were swollen with blurred cristae, while some of the mitochondria were similar to the vacuole. The number of autophagosomes was found to be increased. The arrangement of the myocardium in the 3‐methyladenine group and the QSYQ group was slightly disordered and loose. Some mitochondria were swollen, while some were vacuole‐like, and the number of autophagosomes was reduced (Figure 5).

### 3.5 Effect of QSYQ on Beclin‐1, LC3B and p62 mRNA expression in rat myocardium

**FIGURE 6:**
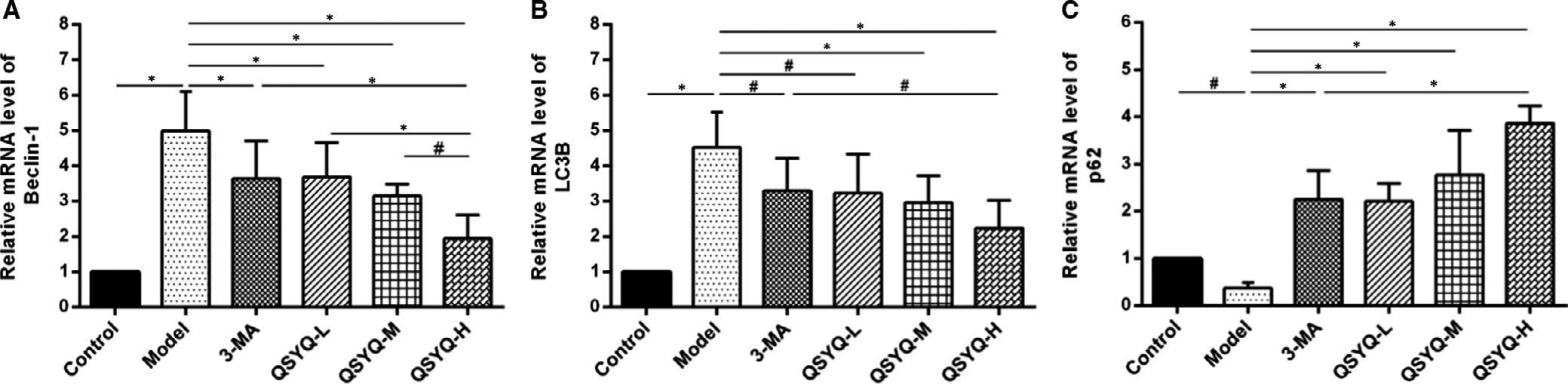
Effect of QiShenYiQi Pill (QSYQ) on mRNA expression of Beclin‐1, LC3B and p62 in rats. A, The Beclin‐1 mRNA expression for each group. B, The LC3B mRNA expression for each group. C, The p62 mRNA expression for each group. Data are expressed as mean ± SD. *P < 0.01, ^#^ *P *< 0.05

Compared with the control group, the expression of Beclin‐1 and LC3B mRNA in the myocardium of the model group increased (*P* < 0.01), while the expression of p62 mRNA decreased (*P* < 0.05). Compared with the model group, the expression of Beclin‐1 and LC3B mRNA in the 3‐methyladenine group and the QSYQ group decreased (*P* < 0.01 or *P* < 0.05), while the expression of p62 mRNA increased (*P* < 0.01). This result suggested that 3‐methyladenine and QSYQ decreased the expression of autophagy‐related genes in rats, by inhibiting the myocardial excessive autophagy. The effect of QSYQ was dose‐dependent as the inhibitory effect of high‐dose QSYQ was more significant (*P* < 0.01) (Figure 6).

### 3.6 Effect of QSYQ on the expression of myocardial autophagy‐related proteins in rats

**FIGURE 7:**
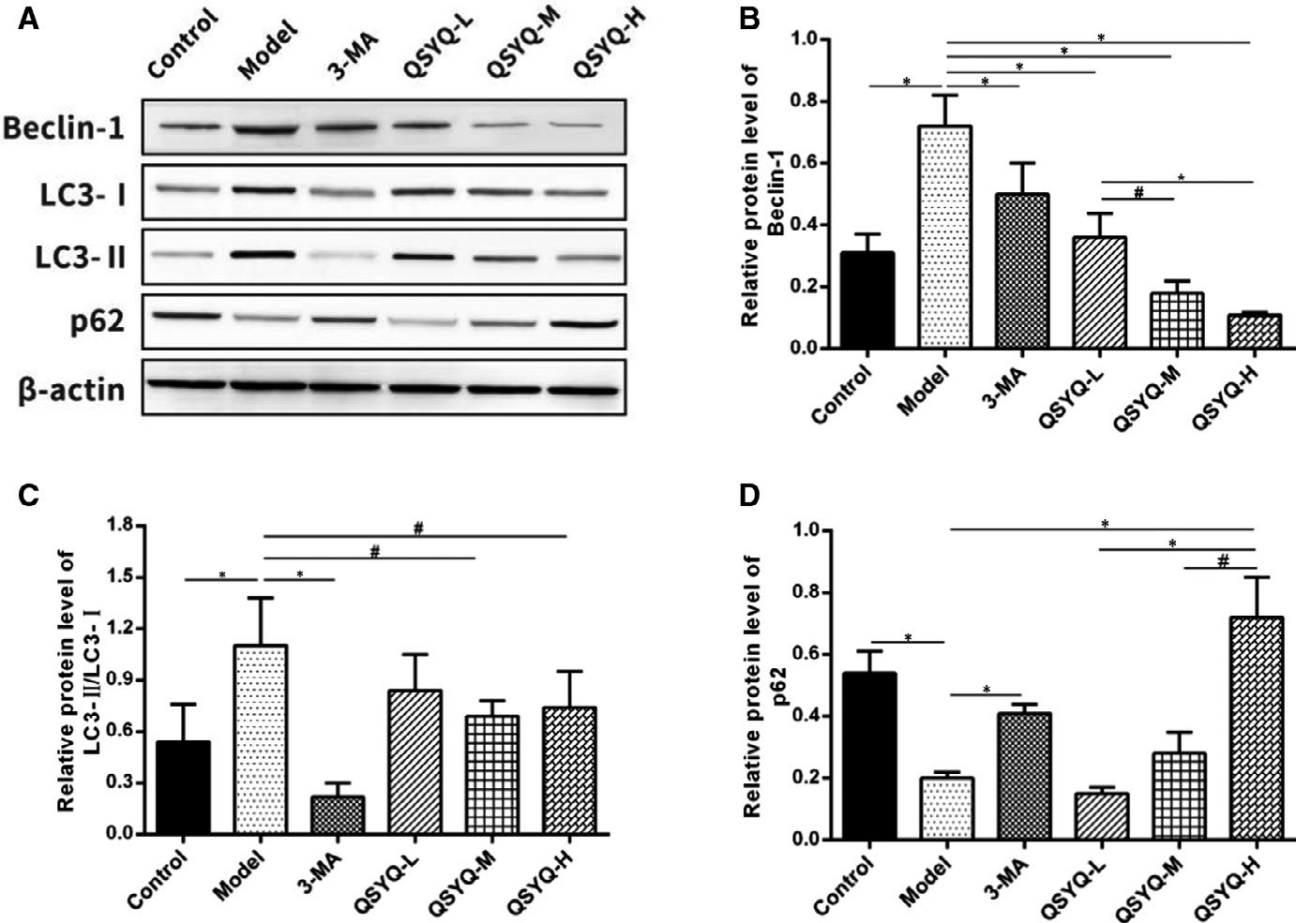
Effect of QiShenYiQi Pill (QSYQ) on the protein expression of Beclin‐1, LC3‐II, LC3‐I and p62 in rats. A, Beclin‐1, LC3‐II, LC3‐I and p62 expression in the myocardium of rats in each group was detected by Western blot. B, The relative protein level of Bcelin‐1 in the myocardium. C, The relative protein level of LC3‐II/LC3‐I in the myocardium. D, The relative protein level of p62 in the myocardium. Data are expressed as mean ± SD. *P < 0.01, ^#^ *P *< 0.05

Compared with the control group, the expression of Beclin‐1 and LC3‐II/LC3‐I in the model group was up‐regulated (*P* < 0.01), while the expression of p62 was down‐regulated (*P* < 0.01). This indicated that excessive autophagy occurred in the myocardium of the model group. Compared with the model group, the expression of Beclin‐1 and LC3‐II/LC3‐I was down‐regulated in the 3‐methyladenine group and the QSYQ group (*P* < 0.01 or *P* < 0.05) but p62 exhibited up‐regulated expressions (*P* < 0.01). The results suggested that 3‐methyladenine and QSYQ inhibited excessive autophagy in rat myocardium and the effect of QSYQ high‐dose group on autophagy was more significant (Figure 7).

### 3.7 Effect of QSYQ on PI3K/Akt‐mTOR pathway in rat myocardium

**FIGURE 8:**
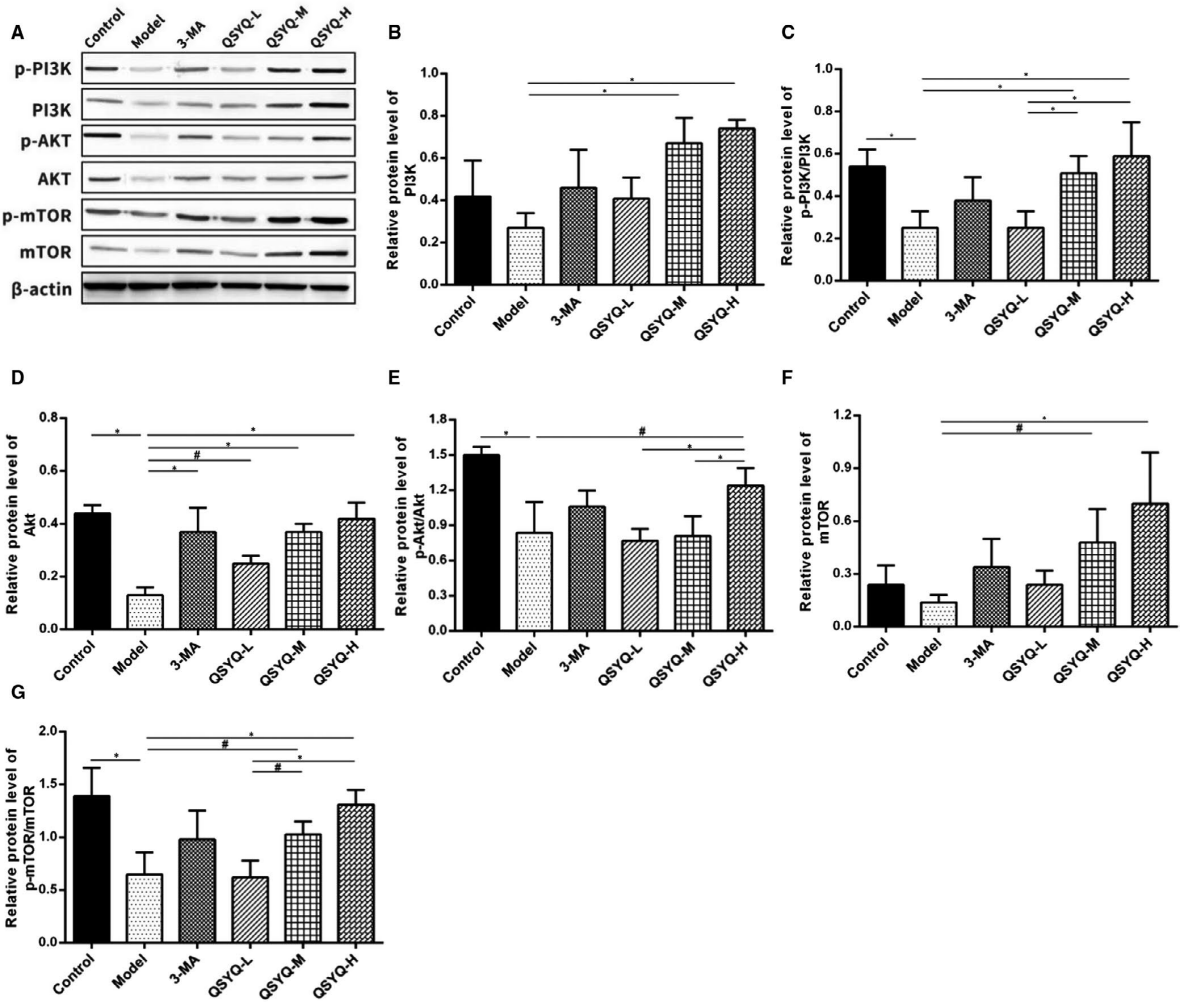
Effect of QiShenYiQi Pill (QSYQ) on the main molecules of PI3K / Akt‐mTOR pathway. A, The protein in the myocardium of rats in each group was detected by Western blot. B, The relative protein level of PI3K in the myocardium. C, The relative protein level of p‐PI3K/PI3K in the myocardium. D, The relative protein level of AKT in the myocardium. E, The relative protein level of p‐AKT/AKT in the myocardium. F, The relative protein level of mTOR in the myocardium. G, The relative protein level of pmTOR/ mTOR in the myocardium. Data are expressed as mean ± SD. *P<0.01, #*P*<0.05.

Compared with the control group, the myocardial Akt protein, p‐PI3K/PI3K, p‐Akt/Akt and p‐mTOR/mTOR in the model group decreased (*P* < 0.01). Additionally, the differences in myocardial PI3K protein, mTOR protein, p‐PI3K/PI3K, p‐Akt/Akt and p‐mTOR/mTOR in the 3‐methyladenine group were not statistically significant (*P* > 0.05) compared to the model group, but Akt protein expression was significantly up‐regulated (*P* < 0.01). The myocardial PI3K protein, Akt protein, mTOR protein, p‐PI3K/PI3K, p‐Akt/Akt and p‐mTOR/mTOR increased (*P* < 0.01 or *P* < 0.05) in QSYQ group in a dose‐dependent manner. This result indicated that QSYQ effectively inhibited the excessive autophagy in rat myocardium by regulating the expression of PI3K/AKT‐mTOR pathway‐related proteins (Figure 8).

## 4 DISCUSSION

Myocardial fibrosis is mainly characterized by abnormal deposition of extracellular matrix and rigidity of myocardial movement, leading to changes in myocardial structure and cardiac systolic dysfunction.^28^, ^29^ Reactive myocardial fibrosis was triggered by pressure loaded, and ischaemia or metabolic injury could expand and thicken the myocardial muscle bundles and its surrounding thin fibre tissues. In contrast, the reparative myocardial fibrosis triggered by necrosis of myocardial cells could develop scars by replacing small lesions of myocardial cell necrosis.^30^ Research fonts have shown that myocardial fibrosis is a potential risk factor for myocarditis and dilated cardiomyopathy resulting in heart failure.^31^, ^32^ Therefore, the effective control of myocardial fibrosis may provide an insight into the prevention and treatment of cardiovascular diseases such as myocarditis, dilated cardiomyopathy and heart failure. In the current study, the administration of QSYQ reduced HMI, LVMI, LVEDD, and LVESD and enhanced LVEF and LVFS. Moreover, the myocardial inflammation histological score and myocardial collagen volume fraction were also declined. This phenomenon may attribute to the fact that QSYQ exhibits an effect on reparative myocardial fibrosis in a dose‐dependent manner. It should be noted that the effect of QSYQ high‐dose group on reparative myocardial fibrosis was more significant.

The occurrence of myocardial fibrosis includes inflammation, mitochondrial damage, apoptosis, fibroblast proliferation and myofibroblast phenotype conversion, but the underlying mechanisms are still uncovered.^33^ Autophagy is closely related to myocardial fibrosis, the excessive activation of autophagy may induce myocardial collagen deposition and fibrosis and exacerbate cardiac dysfunction such as decreased heart function and heart failure.^33^, ^34^, ^35^ Autophagy is a highly catabolic process of the degradation or recycling of damaged or unnecessary proteins and organelles mediated by lysosomes, which is essential to maintain cellular stability.^35^ Inhibiting myocardial excessive autophagy can restrain the phenotype conversion of fibroblasts into myofibroblasts and improve myocardial fibrosis.^36^ Autophagy is best detected by the electron microscopy and is characterized by a double‐layer or single‐layer membrane in myocardial cells.^37^ Beclin‐1, LC3B and p62, the most commonly used autophagy‐related markers are the three most important proteins involved in the process of autophagy.^38^ Among them, Beclin‐1 is the initiation factor in the process of autophagy and the expression level of Beclin‐1 is closely related to autophagy activity.^39^ LC3B has two subtypes, including LC3‐I and LC3‐II. The conversion of LC3‐I into LC3‐II is a key procedure in the formation of autophagosomes. P62 has been considered as one of the selective substrates of LC3B. When the autophagy programme starts, p62 first binds to the ubiquitous protein, then forms a complex with LC3‐II and finally degrades through autophagolysosome.^40^ In this study, the myocardium in QSYQ group was slightly disordered and loosen compared with the model group and a few mitochondria were swollen with some visible vacuoles. The number of autophagosomes was also observed to be reduced. The expression of Beclin‐1 and LC3‐II/LC3‐I was down‐regulated, while the expression of p62 was up‐regulated. This indicated that QSYQ could inhibit excessive autophagy in rat myocardium in a dose‐dependent manner.

The PI3K/Akt‐mTOR pathway is the most critical upstream signalling pathway associated with the regulation of autophagy.^41^ In this pathway, when the cells are stimulated by the surrounding environment, the activated PI3K‐I activates Akt with the help of phosphatidylinositol lipid‐dependent protein kinase and inhibits the TSC1/2 complex to activate mTOR and inhibit autophagy.^42^, ^43^ The PI3K/Akt‐mTOR pathway is associated with the occurrence of myocardial fibrosis and has a regulatory effect on the proliferation and migration of cardiac fibroblasts.^44^ Accumulating evidence has demonstrated that blocking autophagy could enhance the TGF‐β1 induced myocardial fibrosis.^45^ It has been also documented that the activation of the PI3K/Akt‐mTOR pathway could inhibit myocardial fibrosis, hypertrophic or dilated cardiomyopathy by blocking myocardial fibrosis and then improving diastolic function.^46^ Besides, in the myocardial ischaemia/reperfusion (I/R) model, basic fibroblast growth factor could inhibit myocardial excessive autophagy by activating the PI3K/Akt‐mTOR pathway thereby improving the cardiac function.^47^ In this study, it was found that QSYQ could increase the ratios of p‐PI3K/PI3K, p‐Akt/Akt and p‐mTOR/mTOR in rat myocardium in a dose‐dependent manner, suggesting that QSYQ can effectively inhibit excessive autophagy in rat myocardium by regulating the expression of PI3K/Akt‐mTOR pathway‐related proteins.

## 5 CONCLUSION

In summary, the QSYQ compound could inhibit excessive myocardial autophagy by regulating the PI3K/Akt‐mTOR pathway to exert an effect on reparative myocardial fibrosis and can be a potential therapeutic approach to treat the cardiovascular diseases such as myocarditis and dilated cardiomyopathy.

## CONFLICT OF INTEREST

The authors declare no competing interests.

## AUTHOR CONTRIBUTION

**Shichao Lv:** Data curation (lead); Formal analysis (lead); Funding acquisition (lead); Writing‐original draft (lead); Writing‐review & editing (lead). **Peng Yuan:** Investigation (equal); Methodology (equal). **Jianping Dong:** Investigation (equal). **Chunmiao lu:** Data curation (equal). **Meng Li:** Investigation (equal). **Fan Qu:** Data curation (equal). **Yaping Zhu:** Formal analysis (supporting); Writing‐review & editing (supporting). **Yuan Zhuo:** Funding acquisition (supporting). **Junping zhang:** Conceptualization (lead); Project administration (lead); Supervision (lead).

## Data Availability

Data can be accessed by emailing the corresponding authors.

## References

[1] Gyöngyösi M , Winkler J , Ramos I , et al. Myocardial fibrosis: biomedical research from bench to bedside. Eur J Heart Fail. 2017;19(2):177‐191. 28157267 10.1002/ejhf.696 PMC5299507

[2] Talman V , Ruskoaho H . Cardiac fibrosis in myocardial infarction—from repair and remodeling to regeneration. Cell Tissue Res. 2016;365(3):563‐581. 27324127 10.1007/s00441-016-2431-9 PMC5010608

[3] Knaapen P , Götte MJW , Paulus WJ , et al. Does myocardial fibrosis hinder contractile function and perfusion in idiopathic dilated cardiomyopathy? PET and MR imaging study. Radiology. 2006;240(2):380‐388. 16864667 10.1148/radiol.2402051038

[4] Rubiś P , Totoń‐Żurańska J , Wiśniowska‐Śmiałek S , et al. Relations between circulating microRNAs (miR‐21, miR‐26, miR‐29, miR‐30 and miR‐133a), extracellular matrix fibrosis and serum markers of fibrosis in dilated cardiomyopathy. Int J Cardiol. 2017;231:201‐206. 27889210 10.1016/j.ijcard.2016.11.279

[5] Komajda M , Jais JP , Reeves F , et al. Factors predicting mortality in idiopathic dilated cardiomyopathy. Eur Heart J. 1990;11(9):824‐831. 2226508 10.1093/oxfordjournals.eurheartj.a059803

[6] Kaya Z , Katus HA . Role of autoimmunity in dilated cardiomyopathy. Basic Res Cardiol. 2010;105(1):7‐8. 19826854 10.1007/s00395-009-0069-4

[7] Ciháková D , Sharma RB , Fairweather D , et al. Animal models for autoimmune myocarditis and autoimmune thyroiditis. Methods Mol Med. 2004;102:175‐193. 15286386 10.1385/1-59259-805-6:175

[8] Bracamonte‐Baran W , Čiháková D . Cardiac autoimmunity: myocarditis. Adv Exp Med Biol. 2017;1003:187‐221. 28667560 10.1007/978-3-319-57613-8_10 PMC5706653

[9] Lin SQ , Wei XH , Huang P , et al. QiShenYiQi Pills® prevent cardiac ischemia‐reperfusion injury via energy modulation. Int J Cardiol. 2013;168(2):967‐974. 23168012 10.1016/j.ijcard.2012.10.042

[10] Chen JR , Wei J , Wang LY , et al. Cardioprotection against ischemia/reperfusion injury by QiShenYiQi Pill® via ameliorate of multiple mitochondrial dysfunctions. Drug Des Devel Ther. 2015;9:3051‐3066. 10.2147/DDDT.S82146 PMC4474392 26109848

[11] Lv S , Wu M , Li M , et al. Effect of QiShenYiQi Pill on myocardial collagen metabolism in rats with partial abdominal aortic coarctation. Evid Based Complement Alternat Med. 2015;2015:1‐10. 10.1155/2015/415068 PMC4377429 25861361

[12] Chen Y‐Y , Li Q , Pan C‐S , et al. QiShenYiQi Pills, a compound in Chinese medicine, protects against pressure overload‐induced cardiac hypertrophy through a multi‐component and multi‐target mode. Sci Rep. 2015;5:11802. 26136154 10.1038/srep11802 PMC4488877

[13] Li Y‐C , Liu Y‐Y , Hu B‐H , et al. Attenuating effect of post‐treatment with QiShen YiQi Pills on myocardial fibrosis in rat cardiac hypertrophy. Clin Hemorheol Microcirc. 2012;51(3):177‐191. 22240383 10.3233/CH-2011-1523

[14] Li C , Wang Y , Qiu QI , et al. Qishenyiqi protects ligation‐induced left ventricular remodeling by attenuating inflammation and fibrosis via STAT3 and NF‐κB signaling pathway. PLoS One. 2014;9(8):e104255. 25122164 10.1371/journal.pone.0104255 PMC4133204

[15] Wang J , Lu L , Wang Y , et al. Qishenyiqi Dropping Pill attenuates myocardial fibrosis in rats by inhibiting RAAS‐mediated arachidonic acid inflammation. J Ethnopharmacol. 2015;176:375‐384. 26590099 10.1016/j.jep.2015.11.023

[16] JianXin C , Xue XU , ZhongFeng LI , et al. Qishen Yiqi Drop Pill improves cardiac function after myocardial ischemia. Sci Rep. 2016;6:24383. 27075394 10.1038/srep24383 PMC4830957

[17] Tong JY , Xu YJ , Bian YP , et al. Effect and mechanism of Qishen Yiqi Pills on adriamycin‐ induced cardiomyopathy in mice. Chin J Nat Med. 2013;11(5):514‐518. 24359776 10.1016/S1875-5364(13)60093-X

[18] Tang D‐X , Zhao H‐P , Pan C‐S , et al. QiShenYiQi Pills, a Compound Chinese Medicine, Ameliorates Doxorubicin‐Induced Myocardial Structure Damage and Cardiac Dysfunction in Rats. Evid Based Complement Alternat Med. 2013;2013:480597. 23533487 10.1155/2013/480597 PMC3600323

[19] Lv S , Wu M , Li M , et al. Effect and mechanism of QiShenYiQi Pill on experimental autoimmune myocarditis rats. Med Sci Monit. 2016;22:752‐756. 26946470 10.12659/MSM.895655 PMC4784548

[20] Lv S‐C , Wu M , Li M , et al. Effect of QiShenYiQi pill on myocardial collagen metabolism in experimental autoimmune myocarditis rats. Biomed Pharmacother. 2017;88:894‐901. 28178619 10.1016/j.biopha.2017.01.096

[21] Zhang S , Wang H , Li L , et al. Qishen Yiqi Drop Pill, a novel compound Chinese traditional medicine protects against high glucose‐induced injury in cardiomyocytes. J Cell Mol Med. 2019;23:6393‐6402. 31278860 10.1111/jcmm.14527 PMC6714141

[22] Kodama M , Matsumoto Y , Fujiwara M , et al. A novel experimental model of giant cell myocarditis induced in rats by immunization with cardiac myosin fraction. Clin Immunol Immunopathol. 1990;57(2):250‐262. 2208806 10.1016/0090-1229(90)90039-s

[23] Liu W , Li W‐M , Gao C , et al. Effects of atorvastatin on the Th1/Th2 polarization of ongoing experimental autoimmune myocarditis in Lewis rats. J Autoimmun. 2005;25:258‐263. 16242301 10.1016/j.jaut.2005.06.005

[24] Wu Y‐T , Tan H‐L , Shui G , et al. Dual role of 3‐methyladenine in modulation of autophagy via different temporal patterns of inhibition on Class I and III Phosphoinositide 3‐kinase. J Biol Chem. 2010;285:10850‐10861. 20123989 10.1074/jbc.M109.080796 PMC2856291

[25] Shuyun X , Bian R , Chen X . Experimental Methodology of Pharmacology (Third Edition). Beijing, China: People's Medical Publishing House; 2002:200‐223.

[26] Guifeng Z , Mao J , Liu W , et al. Qishen Yiqi dripping pill inhibited myocardial fibrosis after myocardial infarction in rats and its effects on TGF‐β‐1/Smads pathway. Zhongguo Zhong Xi Yi Jie He Za Zhi. 2017;37:1466‐1470.

[27] Matsumoto Y , Tsukada Y , Miyakoshi A , et al. C protein‐induced myocarditis and subsequent dilated cardiomyopathy: rescue from death and prevention of dilated cardiomyopathy by chemokine receptor DNA therapy. J Immunol. 2004;173(5):353535‐353541. 10.4049/jimmunol.173.5.3535 15322218

[28] Heymans S , González A , Pizard A , et al. Searching for new mechanisms of myocardial fibrosis with diagnostic and/or therapeutic potential. Eur J Heart Fail. 2015;17:764‐771. 26126780 10.1002/ejhf.312

[29] Wang L , Yuan D , Zheng J , et al. Chikusetsu saponin IVa attenuates isoprenaline‐induced myocardial fibrosis in mice through activation autophagy mediated by AMPK/mTOR/ULK1 signaling. Phytomedicine. 2019;58:152764. 31005723 10.1016/j.phymed.2018.11.024

[30] Gonzalez A , Schelbert EB , Diez J , et al. Myocardial interstitial fibrosis in heart failure: biological and translational perspectives. J Am Coll Cardiol. 2018;71(15):1696‐1706. 29650126 10.1016/j.jacc.2018.02.021

[31] Guo Y , Wu W , Cen Z , et al. IL‐22‐producing Th22 cells play a protective role in CVB3‐induced chronic myocarditis and dilated cardiomyopathy by inhibiting myocardial fibrosis. Virol J. 2014;11:230. 25547181 10.1186/s12985-014-0230-z PMC4304148

[32] Daskalopoulos EP , Dufeys C , Bertrand L , et al. AMPK in cardiac fibrosis and repair: Actions beyond metabolic regulation. J Mol Cell Cardiol. 2016;91:188‐200. 26772531 10.1016/j.yjmcc.2016.01.001

[33] Wu R‐N , Yu T‐Y , Zhou J‐C , et al. Targeting HMGB1 ameliorates cardiac fibrosis through restoring TLR2‐mediated autophagy suppression in myocardial fibroblasts. Int J Cardiol. 2018;267:156‐162. 29957254 10.1016/j.ijcard.2018.04.103

[34] Hang P , Zhao J , Su Z , et al. Choline inhibits ischemia‐reperfusion‐induced cardiomyocyte autophagy in rat myocardium by activating Akt/mTOR signaling. Cell Physiol Biochem. 2018;45(5):2136‐2144. 29533930 10.1159/000488049

[35] Liang B , Xiao T , Long J , et al. Hydrogen sulfide alleviates myocardial fibrosis in mice with alcoholic cardiomyopathy by downregulating autophagy. Int J Mol Med. 2017;40(6):1781‐1791. 29039471 10.3892/ijmm.2017.3191 PMC5716447

[36] Gupta SS , Zeglinski MR , Rattan SG , et al. Inhibition of autophagy inhibits the conversion of cardiac fibroblasts to cardiac myofibroblasts. Oncotarget. 2016;7(48):78516‐78531. 27705938 10.18632/oncotarget.12392 PMC5346657

[37] Mizushima N . Methods for monitoring autophagy. Int J Biochem Cell Biol. 2004;36(12):2491‐2502. 15325587 10.1016/j.biocel.2004.02.005

[38] Klionsky DJ , Abdelmohsen K , Abe A , et al. Guidelines for the use and interpretation of assays for monitoring autophagy. Autophagy. 2016;12(1):1‐222. 26799652 10.1080/15548627.2015.1100356 PMC4835977

[39] Cao Y , Klionsky DJ . Physiological functions of Atg6/Beclin 1: a unique autophagy‐related protein. Cell Res. 2007;17(10):839‐849. 17893711 10.1038/cr.2007.78

[40] Komatsu M , Ichimura Y . Physiological significance of selective degradation of p62 by autophagy. FEBS Lett. 2010;584(7):1374‐1378. 20153326 10.1016/j.febslet.2010.02.017

[41] Yue LU , Ailin W , Jinwei Z , et al. PSORI‐CM02 ameliorates psoriasis in vivo and in vitro by inducing autophagy via inhibition of the PI3K/Akt/mTOR pathway. Phytomedicine. 2019;64:153054. 31401494 10.1016/j.phymed.2019.153054

[42] Aoki M , Fujishita T . Oncogenic roles of the PI3K/AKT/mTOR axis. Curr Top Microbiol Immunol. 2017;407:153‐189. 28550454 10.1007/82_2017_6

[43] Brown JS , Banerji U . Maximising the potential of AKT inhibitors as anti‐cancer treatments. Pharmacol Ther. 2017;172:101‐115. 27919797 10.1016/j.pharmthera.2016.12.001 PMC6143165

[44] Yang W , Wu Z , Yang KE , et al. BMI1 promotes cardiac fibrosis in ischemia‐induced heart failure via the PTEN‐PI3K/Akt‐mTOR signaling pathway. Am J Physiol Heart Circ Physiol. 2019;316(1):H61‐H69. 30359076 10.1152/ajpheart.00487.2018

[45] Yuan Y , Zhang Y , Han X , et al. Relaxin alleviates TGFbeta1‐induced cardiac fibrosis via inhibition of Stat3‐dependent autophagy. Biochem Biophys Res Commun. 2017;493(3):1601‐1607. 28942152 10.1016/j.bbrc.2017.09.110

[46] De Los SS , Palma‐Flores C , Zentella‐Dehesa A , et al. (‐)‐Epicatechin inhibits development of dilated cardiomyopathy in delta sarcoglycan null mouse. Nutr Metab Cardiovasc Dis. 2018;28(11):1188‐1195. 30143409 10.1016/j.numecd.2018.06.019

[47] Wang Z‐G , Wang Y , Huang Y , et al. bFGF regulates autophagy and ubiquitinated protein accumulation induced by myocardial ischemia/reperfusion via the activation of the PI3K/Akt/mTOR pathway. Sci Rep. 2015;5:9287. 25787015 10.1038/srep09287 PMC4365411

